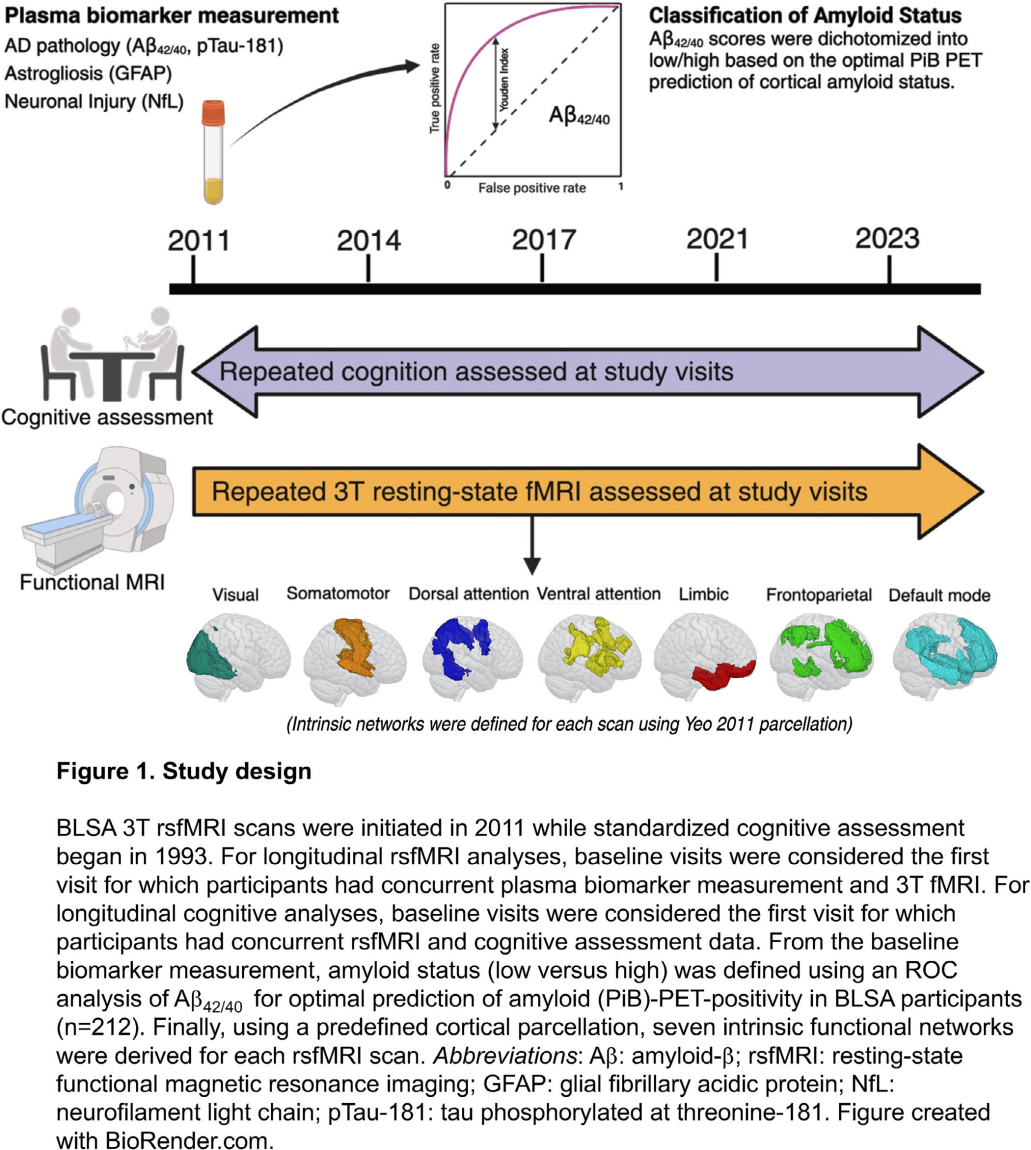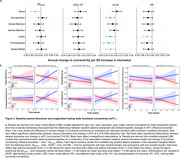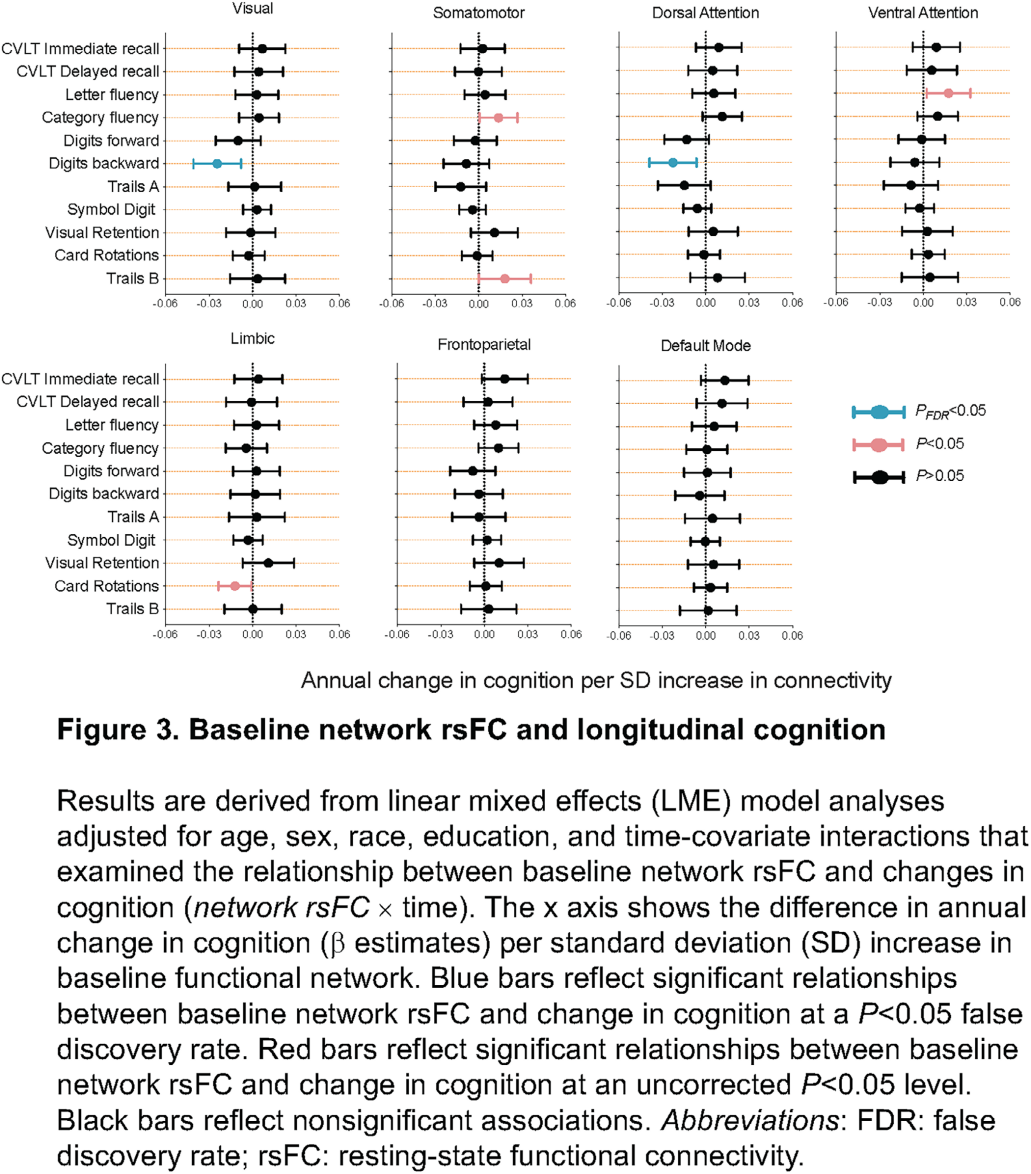# Plasma ADRD biomarkers predict longitudinal declines in intra‐network functional brain connectivity, and baseline functional connectivity predicts longitudinal cognition

**DOI:** 10.1002/alz.092515

**Published:** 2025-01-09

**Authors:** Heather E Dark, Andrea T Shafer, Jenifer Cordon, Yang An, Alexandria Lewis, Abhay Moghekar, Bennett A. Landman, Susan M. Resnick, Keenan A. Walker

**Affiliations:** ^1^ National Institute on Aging, National Institutes of Health, Baltimore, MD USA; ^2^ Johns Hopkins University School of Medicine, Baltimore, MD USA; ^3^ Department of Electrical and Computer Engineering, Vanderbilt University, Nashville, TN USA; ^4^ Department of Radiology & Radiological Sciences, Vanderbilt University Medical Center, Nashville, TN USA

## Abstract

**Background:**

AD is defined by cortical amyloid‐β (Aβ), tau neurofibrillary tangles, and neurodegeneration, pathological processes which may contribute to cognitive decline by altering large scale functional brain networks. To test this hypothesis, we examined whether plasma biomarkers of AD pathology (Aβ_42/40_, phosphorylated tau [pTau‐181]), astrogliosis (glial fibrillary acidic protein [GFAP]), and neuronal injury (neurofilament light chain [NfL]) related to longitudinal changes in resting‐state functional connectivity (rsFC) in cognitively unimpaired participants from the Baltimore Longitudinal Study of Aging.

**Method:**

Baseline plasma biomarkers were measured with Quanterix SIMOA assays. Functional connectivity (3T resting‐state fMRI) was derived using a predefined cortical parcellation mask from which intra‐network connectivity from seven functional networks was extracted for each participant. Amyloid status (positive/negative) was defined using plasma Aβ_42/40_ (Figure 1). Linear mixed effects models adjusted for age, sex, race, education, gray matter volume, and time‐covariate interactions were used to determine whether 1) baseline plasma biomarkers predicted longitudinal changes in rsFC, 2) the magnitude of the biomarker‐related rsFC changes differed by amyloid status, and 3) rsFC predicted longitudinal changes in cognition.

**Result:**

Longitudinal connectivity analyses (mean age±SD=65.49±16.17) included 490 participants (1190 visits; mean follow‐up time=4.31±1.68 years). Higher Aβ_42/40_, GFAP, and NfL were associated with faster declines in rsFC within several networks (P‐range=0.01‐0.04; Figure 2). Overall, plasma biomarker‐rsFC associations differed by amyloid status (P‐range=0.01‐0.045). Among amyloid‐positive participants, lower levels of Aβ_42/40_, and higher levels of GFAP, and NfL (Figure 2) were associated with faster declines in rsFC in the visual, dorsal and ventral attention, limbic, and frontoparietal networks (P range=<0.002‐0.04). There were no statistically significant associations between plasma biomarkers and rsFC change among amyloid‐negative participants. Among 760 participants with at least one rsFC scan (mean age±SD=67.21±14.93; 1550 visits, follow‐up time=3.94±1.60 years), we found that baseline rsFC in several networks predicted changes in cognition, e.g., working memory, verbal fluency, and visuospatial abilities (P range=0.02‐0.049; Figure 3).

**Conclusion:**

Among cognitively normal individuals, plasma biomarkers of Aβ_42/40_, astrogliosis, and neuronal injury are associated with future intra‐network functional brain changes, particularly in the context of elevated amyloid. Hypo‐ and hyper‐ intra‐network connectivity may drive changes in cognitive performance.